# A pediatric case of congenital stromal corneal dystrophy caused by the novel variant c.953del of the *DCN* gene

**DOI:** 10.1038/s41439-023-00239-8

**Published:** 2023-03-24

**Authors:** Hazuki Morikawa, Sachiko Nishina, Kaoruko Torii, Katsuhiro Hosono, Tadashi Yokoi, Chika Shigeyasu, Masakazu Yamada, Motomichi Kosuga, Maki Fukami, Hirotomo Saitsu, Noriyuki Azuma, Yuichi Hori, Yoshihiro Hotta

**Affiliations:** 1 Division of Ophthalmology, National Center for Child Health and Development, Tokyo, Japan; 2 Department of Ophthalmology, Toho University Graduate School of Medicine, Tokyo, Japan; 3 Department of Ophthalmology, Hamamatsu University School of Medicine, Hamamatsu, Japan; 4 Department of Ophthalmology, Kyorin University, Tokyo, Japan; 5 Division of Medical Genetics, National Center for Child Health and Development, Tokyo, Japan; 6 Department of Molecular Endocrinology, National Research Institute for Child Health and Development, Tokyo, Japan; 7 Department of Biochemistry, Hamamatsu University School of Medicine, Hamamatsu, Japan

**Keywords:** Diseases, Pathogenesis

## Abstract

We report a 1-year-old girl with congenital stromal corneal dystrophy confirmed by genetic analysis. The ocular phenotype included diffuse opacity over the corneal stroma bilaterally. We performed a genetic analysis to provide counseling to the parents regarding the recurrence rate. Whole exome sequencing was performed on her and her parents, and a novel de novo variant, NM_001920.5: c.953del, p.(Asn318Thrfs*10), in the *DCN* gene was identified in the patient.

Congenital stromal corneal dystrophy (CSCD) is an exceedingly rare disease^1–3^. It is an autosomal dominant trait^4^ and occurs bilaterally^5^. The primary corneal alterations are not associated with previous inflammation or secondary to a systemic disease^1^.

CSCD is caused by mutations in the *decorin* (*DCN*) gene on the long arm of chromosome 12^3,5,6^. Decorin is a member of the small leucine-rich proteoglycan gene family with a variety of binding properties with matrix structural components, including collagens and growth factors^6–9^. Decorin is distributed throughout the body, including the cornea, respiratory system, pancreas, muscle, skin, and ovary. However, no reports have described systemic diseases caused by *DCN* mutations. The only phenotype associated with a mutation in the *DCN* gene is CSCD^9^.

Decorin has a role in the assembly of corneal collagen fibers and supports corneal transparency^3,10–12^. In patients with CSCD with *DCN* mutations, transmission electron microscopy has confirmed disruption of collagen fibers, i.e., separation of the normal lamella of the collagen fibrils by abnormal collagen filaments^1,2,5,13^.

Five families with CSCD have been reported^2,5^, and we recently identified a Japanese child with CSCD as the sixth CSCD pedigree in the world. We also identified a novel mutation in the *DCN* gene. We present the detailed clinical profile of the case with CSCD confirmed by genetic analysis.

The proband was a 1-year 7-month-old girl referred to the Division of Ophthalmology, National Center for Child Health and Development for further examinations and treatment for bilateral corneal opacities. She had no medical history, and her parents were not consanguineous.

At the first visit, she showed no nystagmus and maintained orthophoria without limitations of extraocular movements. Fixation and following behavior of each eye were observed; the visual acuity (VA) using the Teller Acuity Cards II was 20/380 bilaterally. Slit-lamp microscopy showed diffuse opacity bilaterally. The anterior chamber depth was normal, and the corneal diameters were equally normal in both eyes (10.5 mm vertically and 11.0 mm horizontally). The central corneal thickness was 620 μm in the right eye and 640 μm in the left eye (Fig. 1A). The respective intraocular pressures (Eye Care Hand-held Tonometer; Icare TA01i, Helsinki, Finland) were 18 mmHg and 14 mmHg. We conducted a detailed ophthalmic examination with the child under general anesthesia; swept-source optical coherence tomography (SS-OCT) (Topcon, Tokyo, Japan) showed hyperreflective zones consistent with the corneal opacity involving the entire stroma in both eyes (Fig. 1B). Ophthalmoscopy showed no apparent abnormalities in either fundus but poor translucency due to corneal opacity (Fig. 1C). Electroretinography (ERG) (Neuropack, Nihon Koden, Tokyo, Japan) based on the International Society for Clinical Electrophysiology of Vision protocol^14^ showed normal responses (Fig. 1D).

**Fig. 1:**
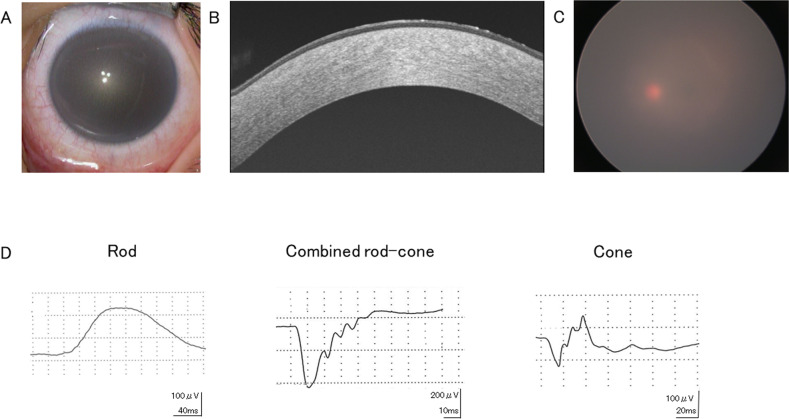
Ophthalmic phenotype of the left eye of the patient. Both eyes had similar findings. **A** The anterior segment has diffuse corneal opacity. **B** An OCT image shows hyperreflective zones consistent with corneal opacity involving the entire stroma in both eyes. **C** A fundus photograph shows poor translucency due to corneal opacity, but no abnormalities are seen. **D** The full-field ERG in the left eye shows normal responses of the combined rod-cone, rods, and cones. The calibrations are shown for each stimulation.

We performed a systemic investigation to eliminate other diseases that cause diffuse corneal opacity in infants, e.g., congenital metabolic disorders and congenital infection. Laboratory analyses of the serum antibodies, treponema pallidum, and TORCH antibodies (IgG, IgM) were negative. No abnormal findings were detected in a lysosomal enzyme activity assay or urinary mucopolysaccharide analysis. Imaging, i.e., electrocardiography, echocardiography, abdominal ultrasonography, and bone X-rays, were negative. We clinically diagnosed congenital corneal dystrophy, although the type was not determined.

Because the parents inquired about potential recurrence in a second child, we performed a genetic analysis. Since our patient was born to healthy parents, the inheritance was suspected to be autosomal recessive or dominant inheritance of a de novo mutation.

To investigate the genetic background, we performed whole exome sequencing (WES) in three family members and analyzed them via autosomal recessive and dominant models. Genomic DNA was extracted from peripheral blood using standard procedures. Exome data processing, variant calling, and variant annotation were performed as previously described^15^ using human GRCh38 as the reference genome. To identify disease-causing variants, we focused on nonsynonymous variants and splice-site variants, which were within 10 base pairs (bp) of the exon‒intron boundaries (±10 bp), and excluded synonymous and noncoding exonic variants from the analysis. We treated common genetic variants (allele frequency >0.01 for recessive variants; >0.001 for dominant variants) in any of the ethnic subgroups found in the following single nucleotide polymorphism databases and in-house exome data (*n* = 218) as putative nonpathogenic sequence alterations using the Genome Aggregation Database (https://gnomad.broadinstitute.org/) and Tohoku Medical Megabank Organization database (4.7KJPN, https://jmorp.megabank.tohoku.ac.jp/).

Potential pathogenic variants detected by WES were validated using Sanger sequencing according to the standard protocol^16^. Sanger sequencing segregation analyses were performed in three family members to investigate cosegregation of the potentially pathogenic variants. The following primer set for the *DCN* gene was used: forward primer 5’-AAGGGCCTCAACATATTTAGAGAAT-3’ and reverse primer 5’-TGCAGTTAGGTTTCCAGTATCTAGC-3’.

Based on the WES data of the affected child (II-1) and parents (I-1 and I-2), we identified a novel heterozygous variant in the patient, NM_001920.5: c.953del, p.(Asn318Thrfs*10), in the *DCN* gene that was de novo in origin.

Since *DCN* was reported as the causative gene for CSCD^1–3,5,6,9^, we further validated the *DCN* variant by Sanger sequencing and cosegregation (Fig. 2). Sanger sequencing indicated that the variant was not in any database described in the methods, indicating that the variant was extremely rare. As a result of the mutation, the asparagine at position 318 becomes threonine. Subsequently, the amino acid sequence changed, and the following 10th codon became a termination codon. This suggests that p.(Asn318Thrfs*10) is deleterious to the helical structure of the protein. This variant was classified as pathogenic^17^ (Supplementary Table 1).

**Fig. 2:**
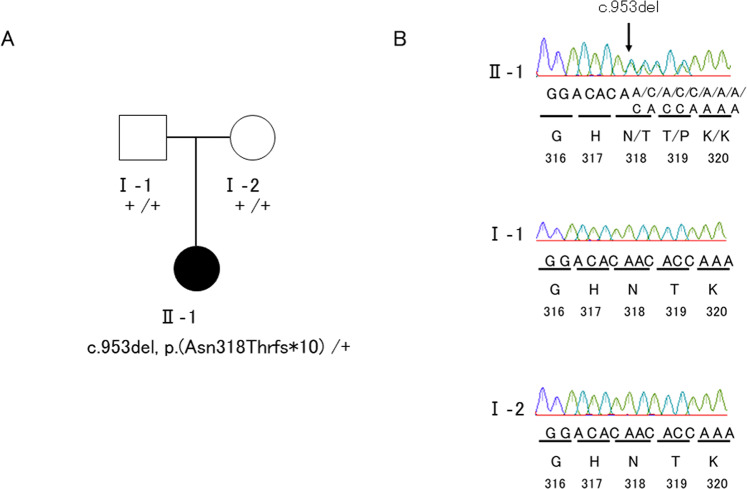
A rare variant of the *DCN* gene. **A** The pedigree of the family. **B** A rare heterozygous *DCN* variant in the affected child: NM_001920.5: c.953del, p.(Asn318Thrfs*10). A heterozygous variant was not identified in the father or mother.

After the definitive diagnosis was made, we performed genetic counseling for the parents. We explained that the recurrence rate in a second child would be generally considered to be low because we identified a de novo variant of the *DCN* gene. However, this could vary depending on gonadal mosaicism and postzygotic DNA^18^. Following genetic counseling, they gave birth to their second child.

The patient was followed up to 3 years of age. The corneal opacity remained unchanged, and the decimal VA was 0.03 bilaterally.

CSCD is a rare hereditary stromal corneal dystrophy caused by *DCN* mutations^1–3,5,6^ that has been reported in only five genetically confirmed pedigrees^2,5^. Detailed descriptions of genetic mutations and clinical characteristics of previous patients and the current patient are shown in Supplementary Table 2.

We documented a de novo novel mutation of the *DCN* gene, NM_001920.5: c.953del, p.(Asn318Thrfs*10), in a Japanese child with CSCD. Interestingly, in the previously reported families with CSCD, the *DCN* mutations were located only in exon 8 of the gene^1–5^, as in the current patient. The structural/functional effects of the mutation on the DCN protein classify it as a truncation mutation of the decorin protein, and the EAR repeat at the C-terminus region was shortened^2,12^. This might affect the collagen-decorin interaction. In a knockout mouse model of CSCD at the C-terminus region, separation of collagen lamellae was seen, and this was seen in CSCD patients^9^. In the model, the truncated *DCN* possibly had a dominant negative effect on wild-type decorin protein that interacted with keratocytes through the signaling receptor on the surface, which resulted in the downregulation of endogenous decorin, biglycan, lumican, and keratocan. Based on the American College of Medical Genetics and Genomics guidelines, the current frameshift mutation was considered pathogenic. This could be because the mutation was located between those of the Norwegian and Belgian families^1,4^, which induced truncation of decorin in the penultimate and longest leucine-rich repeats, known as the EAR repeat region.

The corneal abnormality appeared in infancy, with diffuse corneal opacity and corneal thickening. Although no nystagmus was observed, the measurable VA was 0.03 bilaterally. In a Norwegian report, corneal changes were seen in affected individuals during the first months after birth based on a family interview. The corneal thickness increased, and the VA varied from light perception to 0.6. In the Belgian families, diffuse corneal opacity was observed during early childhood, and the corneal thickness was normal^13^. Compared to that of the Norwegian and Belgian families^1,13^, the age of onset of the current case was comparable. As discussed previously, the current mutation was localized between those of the Norwegian and Belgian cases in the EAR region of decorin, and the phenotype of the current case might be consistent with those cases.

Most previous patients underwent penetrating keratoplasty^1–3,5,13^ between 1 and 44 years of age. The postoperative VA improved or was maintained in many cases, and a few required regrafting because of keratitis, perforation, and corneal opacities. Therefore, penetrating keratoplasty may also have provided good postoperative VA in the current case.

We performed prompt genetic studies that were useful to determine the diagnosis, causative gene, and hereditary form for genetic counseling regarding the birth of a second child and may contribute to treatment decisions.

## HGV Database

The relevant data from this Data Report are hosted at the Human Genome Variation Database at 10.6084/m9.figshare.hgv.3283

### Supplementary information

Supplementary Table 1

Supplementary Table 2

Supplementary Table legend
