# Supplementary material for: A pediatric case of congenital stromal corneal dystrophy caused by the novel variant c.953del of the DCN gene
Source: Hum Genome Var. 2023 Mar 24;10:9. doi: 10.1038/s41439-023-00239-8 (PMC10039048; doi:10.1038/s41439-023-00239-8)
Supplement: Supplementary file 3 — Supplementary Table legend [file 41439_2023_239_MOESM3_ESM.docx]

**Supplementary Table 1** Triple analysis of rare variants from whole-exome sequencing data under the assumption of Mendelian inheritance with complete penetrance (de novo, autosomal dominant, autosomal recessive, and X-linked recessive)

**Supplementary Table 2** The clinical characteristics of previous reports with *DCN* mutations. Five previous cases of CSCD with identified genetic mutations are compared with the present case. There is no uniform association between the genotype and phenotype.
